# How compartments talk: Compartment coupling guides cochlear development

**DOI:** 10.1371/journal.pbio.3003360

**Published:** 2025-09-10

**Authors:** Ippei Kishimoto, Alan G. Cheng

**Affiliations:** Otolaryngology—Head & Neck Surgery, Stanford University School of Medicine, Stanford, California, United States of America

## Abstract

Morphogens cooperate to guide development of the inner ear cochlea, but how do compartments communicate? A recent study in PLOS Biology reveals how planar cell polarity of individual cells is integrated across distinct regional compartments to ensure proper organ morphogenesis.

Precise cellular organization is fundamental to organ development and homeostasis. In the inner ear cochlea, morphogens such as Sonic hedgehog [1] and Wnts [2], cell–cell communication by Notch signaling [3], and planar cell polarity (PCP) [4,5] are known to coordinate to guide development. This spiral organ is longitudinally and radially patterned; however, how different compartments communicate is not clear. In this issue of PLOS Biology, Prakash and colleagues uncover a previously unrecognized mechanism of tissue communication coined “compartment coupling,” where local patterning and cellular behaviors are integrated across compartments [6].

The cochlea is a sensory organ critical for hearing, comprised of a mosaic of sensory and non-sensory cells, forming the organ of Corti as repeating units along the cochlear spiral. The organ of Corti is radially patterned, as are the non-sensory domains flanking it (greater and lesser epithelial ridges, see Fig 1). This precise patterning is crucial for the cochlea to function effectively across a broad spectrum of sound frequencies. As Prakash and colleagues detail, the genesis of this complex structure stems not merely from individual cellular behaviors, but from a broader integrative mechanism—compartment coupling—that binds the various compartments together [6], a phenomenon noted in skin follicles and feather buds [7–9].

**Fig 1:**
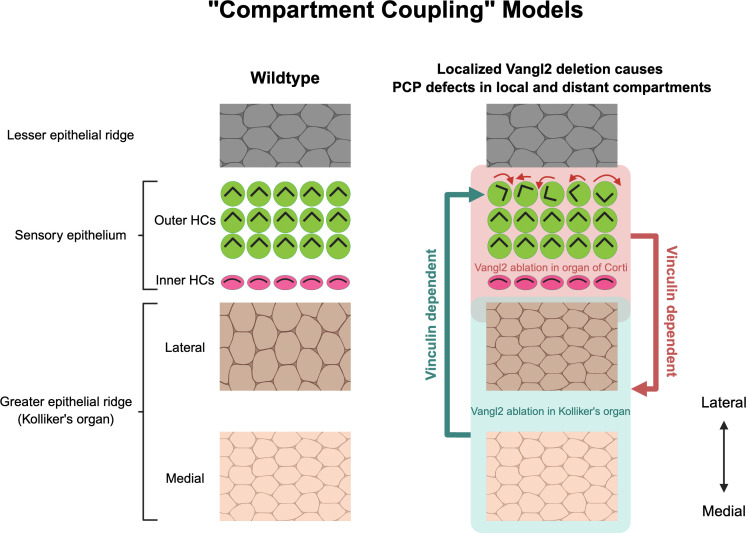
Vangl2 regulates cellular organization in both local and distant compartments through a vinculin-dependent mechanism. The deletion of Vangl2 in either the greater epithelial ridge (GER) or the organ of Corti results in defects in cellular organization, such as the misorientation of hair cells and GER cells. These defects occur in the local and distant compartments, the latter via a vinculin-dependent process termed “compartment coupling”.

Central to their study is the concept of an “adhesion code,” comprised of adhesion molecules expressed in specific compartments, which facilitate communication and coordination among cells during processes like convergent extension and cell specification. The authors showed that the developing organ of Corti is compartmentalized laterally and medially with non-overlapping expression of Cdh1 and Cdh2 (E-cadherin and N-cadherin), respectively. They further showed that this compartmentalization is dependent on Fgfr1, as conditional deletion leads to Cdh1 and Cdh2 co-localization along the medio-lateral axis, indicating a loss of normal compartmentalization. This disruption led to structural anomalies, including sporadic presence of hair cells in non-sensory domains. The findings demonstrate that disruptions in adhesion-based segregation result in structural anomalies within and across cochlear compartments, highlighting the importance of adhesion molecules for proper cochlear development.

One of the most striking observations from the research is the discovery of compartment coupling, where dysfunction in one cellular compartment can exert a non-linear influence on the organization of another compartment (Fig 1). The PCP protein Vangl2 is crucial for the proper orientation of cochlear hair cells and the elongation of the cochlear duct, coined convergent extension [4]. Through targeted ablation of Vangl2 within compartments, the study illustrates that altering cellular organization in one domain (greater epithelial ridge) can disrupt another (lateral organ of Corti) in a non-cell autonomous manner. Furthermore, the study implies that this coupling is mediated by the junctional protein Vinculin, which serves as a scaffold that can transmit forces between compartments and has been shown regulated by PCP signaling [10,11]. As the cochlea is just one of many complex organs that require precise cellular organization across compartments, compartment coupling may represent a fundamental feature of tissue morphogenesis applicable to many developing organs. Moreover, this research highlights the necessity of mechanical interactions, such as those mediated by vinculin, in shaping cellular organization and suggests that similar dynamics might operate in other contexts of organ development.

While these insights are intriguing, the authors acknowledge that the exact molecular pathways involved in ensuring this coupling and the precise nature of the mechanical forces at play require further exploration. It will also be interesting to decipher other components of the adhesion code other than Vangl2 and vinculin. For example, do other PCP core proteins interact with adhesion proteins and similarly participate in compartment coupling? What is the role of morphogens, such as Wnts, in compartment coupling. Since Wnt proteins affect both PCP signaling and beta-catenin which has both transcriptional and structural roles, it is conceivable that they contribute at multiple levels. Furthermore, this study suggests that compartment coupling involves not only mechanical factors but may also incorporate biochemical components. For example, Fgf8, which is expressed at the interface between regions expressing Cdh1 and Cdh2, is known to regulate pillar cell fate [12], suggesting that compartment coupling could also influence cell fate decisions. By illuminating the mechanisms underlying compartment coupling, this study sheds light on the complex choreography of development, emphasizing that the integrity of organ systems relies heavily on the seamless interaction between compartments.

## Abbreviations

**:**
GER: greater epithelial ridge
PCP: planar cell polarity
